# Sex-Specific Association Between Genetic Risk of Psychiatric Disorders and Cardiovascular Diseases

**DOI:** 10.1161/CIRCGEN.124.004685

**Published:** 2024-11-29

**Authors:** Jiayue-Clara Jiang, Kritika Singh, Rachana Nitin, Lea K. Davis, Naomi R. Wray, Sonia Shah

**Affiliations:** Institute for Molecular Bioscience, The University of Queensland, St Lucia, Australia (J.-C.J., N.R.W., S.S.).; Division of Genetic Medicine, Department of Medicine (K.S., R.N., L.K.D.), Vanderbilt University Medical Center, Nashville, TN.; Vanderbilt Genetics Institute (K.S., R.N., L.K.D.), Vanderbilt University Medical Center, Nashville, TN.; Department of Molecular Physiology and Biophysics (L.K.D.), Vanderbilt University Medical Center, Nashville, TN.; Department of Psychiatry and Behavioral Sciences (L.K.D.), Vanderbilt University Medical Center, Nashville, TN.; Departments of Medicine and Biomedical Informatics (L.K.D.), Vanderbilt University Medical Center, Nashville, TN.; Department of Psychiatry, University of Oxford, Warneford Hospital, United Kingdom (N.R.W.).

**Keywords:** cardiovascular diseases, depression, genetic risk score, mental disorders, sex

## Abstract

**BACKGROUND:**

Though epidemiological studies show increased cardiovascular disease (CVD) risks among individuals with psychiatric disorders, findings on sex differences in comorbidity have been inconsistent.

**METHODS:**

This genetic epidemiology study examined the sex-specific association between the genetic risk of 3 psychiatric disorders (major depression [MD], schizophrenia, and bipolar disorder), estimated using polygenic scores (PGSs), and risks of 3 CVDs (atrial fibrillation [AF], coronary artery disease [CAD], and heart failure [HF]) in 345 169 European-ancestry individuals (UK Biobank), with analyses replicated in an independent BioVU cohort (n=49 057). Mediation analysis was conducted to determine whether traditional CVD risk factors could explain any observed sex difference.

**RESULTS:**

In the UK Biobank, a 1-SD increase in PGS_MD_ was significantly associated with the incident risks of all 3 CVDs in females after multiple testing corrections (hazard ratio [HR]_AF-female_=1.04 [95% CI, 1.02–1.06]; *P*=1.5×10^−^^4^; HR_CAD-female_=1.07 [95% CI, 1.04–1.11]; *P*=2.6×10^−^^6^; and HR_HF-female_=1.09 [95% CI, 1.06–1.13]; *P*=9.7×10^−^^10^), but not in males. These female-specific associations remained even in the absence of any psychiatric disorder diagnosis or psychiatric medication use. Although mediation analysis demonstrated that the association between PGS_MD_ and CVDs in females was partly mediated by baseline body mass index, hypercholesterolemia, hypertension, and smoking, these risk factors did not explain the higher risk compared with males. The association between PGS_MD_ and CAD was consistent between females who were premenopausal and postmenopausal at baseline, while the association with AF and HF was only observed in the baseline postmenopausal cohort. No significant association with CVD risks was observed for the PGS of schizophrenia or bipolar disorder. The female-specific positive association of PGS_MD_ with CAD risk was replicated in BioVU.

**CONCLUSIONS:**

Genetic predisposition to MD confers a greater risk of CVDs in females versus males, even in the absence of any depression diagnosis. This study warrants further investigation into whether genetic predisposition to depression could be useful for improving cardiovascular risk prediction, especially in women.

The idea that mental health is linked to cardiovascular health has long been recognized: William Harvey wrote in 1628 “every affection of the mind that is attended with either pain or pleasure, hope or fear, is the cause of an agitation whose influence extends to the heart.”^1^ Modern epidemiological studies find that collectively, individuals with psychiatric disorders, namely, major depression (MD), schizophrenia, and bipolar disorder (BD), have a ≈50% higher odds of developing cardiovascular diseases (CVDs).^2^ The increased CVD risk among individuals with psychiatric disorders may be attributed to a combination of genetic^3^ and nongenetic factors, with the latter including the use of prescribed psychiatric medications, smoking, and social isolation.^4,5^

There are marked sex differences in both the prevalence and clinical presentation of psychiatric disorders and CVDs. Depression is almost twice as prevalent in females than males (lifetime prevalence of 26.1% versus 14.7%),^6^ with women showing augmented symptom severity.^7^ Men show an earlier onset for BD although the prevalence is reported to be equal between sexes for bipolar I disorder but higher in women for bipolar II disorder, which is characterized by depressive episodes.^8^ At the same time, despite a higher lifetime risk of CVD in men (60.3% versus 55.6% in women at an index age of 45 years),^9^ CVD is a leading cause of female deaths,^10^ and yet CVD risk in women remains underestimated and underresearched, leading to underdiagnosis and undertreatment of CVDs in women.^10^

Few studies have investigated the sex differences in the cardiovascular comorbidity of schizophrenia and BD, and observational studies have presented inconsistent findings on the sex-specific association between depression and CVD outcomes.^11^ While some observational studies (N≤3237) have found depression or depressive symptoms to be a risk factor for heart failure (HF) and coronary artery disease (CAD) among women but not in men,^12,13^ a study on a Chinese cohort (N=512 712) found an association between depression and CVD-related mortality in men only.^14^ The variability in findings may be due to differences in sample sizes, unmeasured confounders, follow-up times, sex balance in the cohort, and the criteria for defining depression phenotypes and CVD outcomes. At the same time, observational studies cannot establish causal associations and cannot determine whether this risk is a direct result of medications, which are known to have an adverse cardiometabolic effect,^4^ or other environmental factors in consequence of a diagnosis of depression. Furthermore, although an association between depressive symptoms and increased CAD risk in women aged ≤55 years (but not in women aged >55 years) was previously observed,^13^ it remains unknown how the menopause transition (perimenopause), a period where adverse changes in body composition, lipids, and measures of vascular health occur,^15^ affects the cardiovascular comorbidity among women with a higher risk of depression.

Large genome-wide association studies (GWASs) have been successful at identifying genetic loci associated with disease risk. Novel statistical methods applied to GWAS data are facilitating our understanding of shared biology between diseases, as well as in making causal inferences in disease comorbidity, overcoming some of the caveats of observational studies. Previous genetic analyses, such as summary statistics–based Mendelian randomization analyses, have provided evidence to support a causal effect of depression on CAD and HF.^16^ However, few studies have investigated sex differences using such genetic approaches, with an example being a small-scale study (n=18 385; 50.9% female) that showed a putative causal effect of MD on CAD in females but not males.^17^ To what extent genetic factors contribute to comorbidity between psychiatric disorders and CVDs in different sexes is an outstanding question and one that will better inform CVD prevention strategies. In this study, we used polygenic scores (PGSs) derived from GWAS summary statistics (which estimate an individual’s genetic liability to a disease) to examine the sex-specific association between a higher genetic risk of 3 psychiatric disorders (BD, MD, and schizophrenia) and the incident risk of 3 CVDs [atrial fibrillation (AF), CAD, and HF] in the UK Biobank cohort of 345 169 individuals (definition of incident cases is presented in Figure 1, and summary of characteristics is shown in Table 1). The findings were independently replicated in a BioVU cohort of 49 057 individuals (a summary of characteristics is shown in Table S1).

**Table 1.**
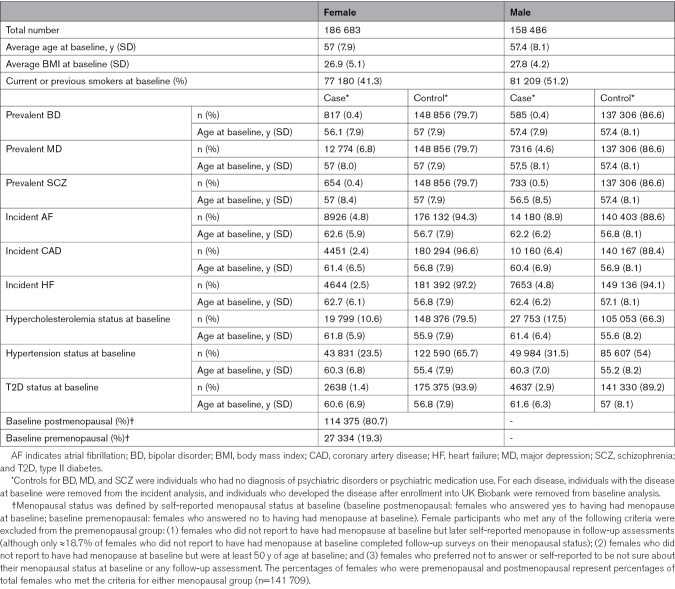
Summary of Characteristics of the UK Biobank Cohort

**Figure 1.**
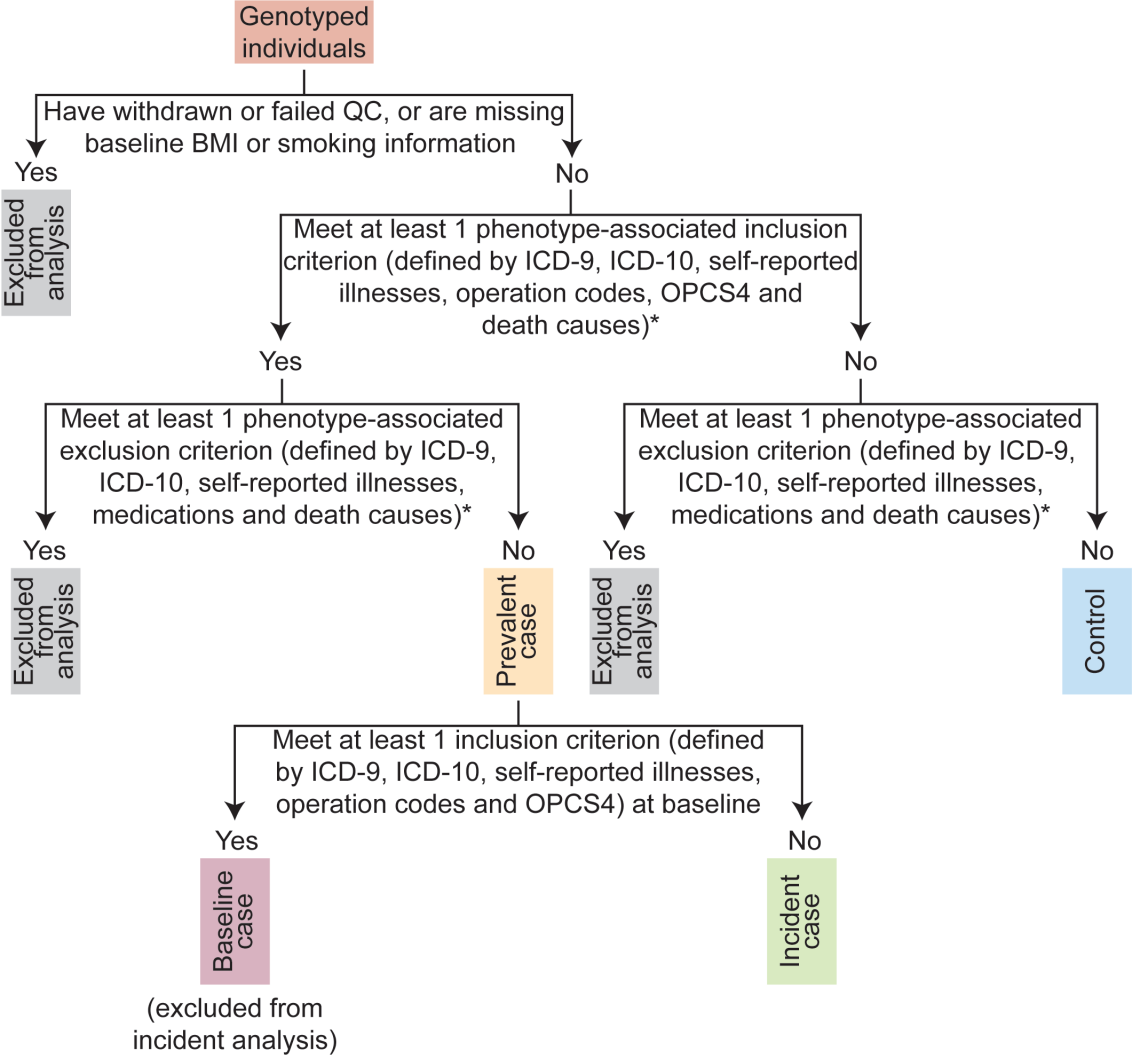
**Flowchart for defining prevalent and incident cardiovascular disease (CVD) cases in the UK Biobank cohort.** *The full inclusion and exclusion criteria for each CVD are presented in Table S4. BMI indicates body mass index; *ICD-10*, *International Classification of Diseases*, *Tenth Edition*; *ICD-9*, *International Classification of Diseases*, *Ninth Edition*; OPCS4, Office of Population Censuses and Surveys, version 4; and QC, quality control.

## Methods

Detailed study methods are presented in Supplemental Methods. For the UK Biobank cohort, consent was collected from all participants by the UK Biobank. The current study was conducted using the UK Biobank Resource under application number 12505. This research is covered by The University of Queensland Human Research Ethics Committee approval (HREC number 2020/HE002938). The BioVU consent form is provided to patients in the outpatient clinic environments at the Vanderbilt University Medical Center. The Vanderbilt University Medical Center Institutional Review Board oversees BioVU and approved this project (IRB#172020). Due to ethics and privacy considerations, requests to access the data set from qualified researchers trained in human subject confidentiality protocols may be sent to UK Biobank and BioVU. The scripts and PGS single-nucleotide polymorphism weights used in the study are available at https://github.com/CNSGenomics/CVD_psych_PRS_Jiang.

## Results

### Increased Genetic Risk of MD Is Associated With an Increase of AF, CAD, and HF Risk in Females

**Figure 2.**
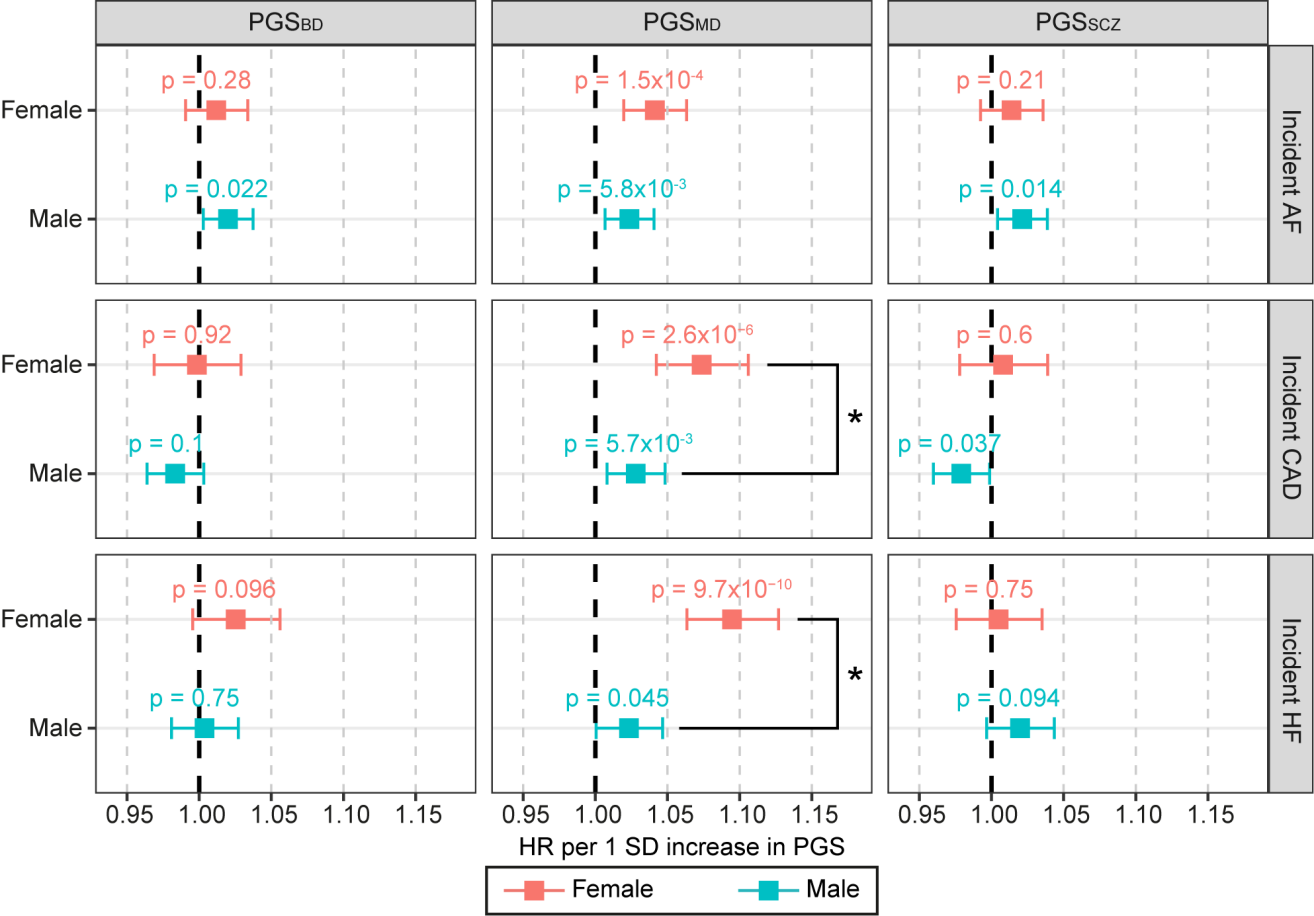
**Change in risk of incident cardiovascular diseases (CVDs) per SD increase in psychiatric disorder polygenic scores (PGSs) in the UK Biobank cohort.** Associations for the sex-stratified cohorts (red: female; blue: male) were estimated with Cox proportional hazards regression models, including body mass index, smoking status, genotyping array, and 20 genetic PCs as covariates. The *x* axis shows the hazard ratio (HR) per SD increase in PGS, with *P* values labeled, and error bars indicate 95% CIs. *A statistically significant difference in the log(HR) values between females and males (2-sided Wald test *P*<0.05). The dark gray line indicates an HR of 1. AF indicates atrial fibrillation; BD, bipolar disorder; CAD, coronary artery disease; HF, heart failure; MD, major depression; and SCZ, schizophrenia.

Sex-stratified Cox proportional hazards regression analysis in the UK Biobank showed that after multiple testing corrections (*P*<2.8×10^−^^3^), a 1-SD increase in PGS_MD_ was significantly associated with increased incident risks of all 3 CVDs in females (hazard ratio [HR]_AF-female_=1.04 [95% CI, 1.02–1.06]; *P*=1.5×10^−^^4^; HR_CAD-female_=1.07 [95% CI, 1.04–1.11]; *P*=2.6×10^−^^6^; and HR_HF-female_=1.09 [95% CI, 1.06–1.13]; *P*=9.7×10^−^^10^) but not in males, and the association with incident CAD and HF was significantly higher in females compared with males (2-sided Wald test *P*=0.016 and 3.2×10^−^^4^, respectively; Figure 2). These associations remained even after correcting for the genetic risks of all 3 CVDs (Figure S1), indicating that PGS_MD_ captured additional risk for CVD over and above, which was captured by the PGSs for all 3 CVDs. No associations between PGS for schizophrenia or BD with CVD risks passed multiple testing corrections in either sex (Figure 2).

Mendelian randomization was performed to test the null association between MD diagnosis and prevalent CVD risks. At *P*<8.3×10^−^^3^ (multiple testing corrections for 6 tests), we found significant evidence to reject the null hypothesis for the association between MD diagnosis and all 3 CVDs in females, as well as the association between MD diagnosis and prevalent CAD in males (Table S2). All of the significant associations showed a positive direction of effect, which indicates that increased risk of MD diagnosis was associated with increased CVD risks.

### The Influence of Menopausal Status on the Association Between Genetic Risk of MD and Incident CVDs

**Figure 3.**
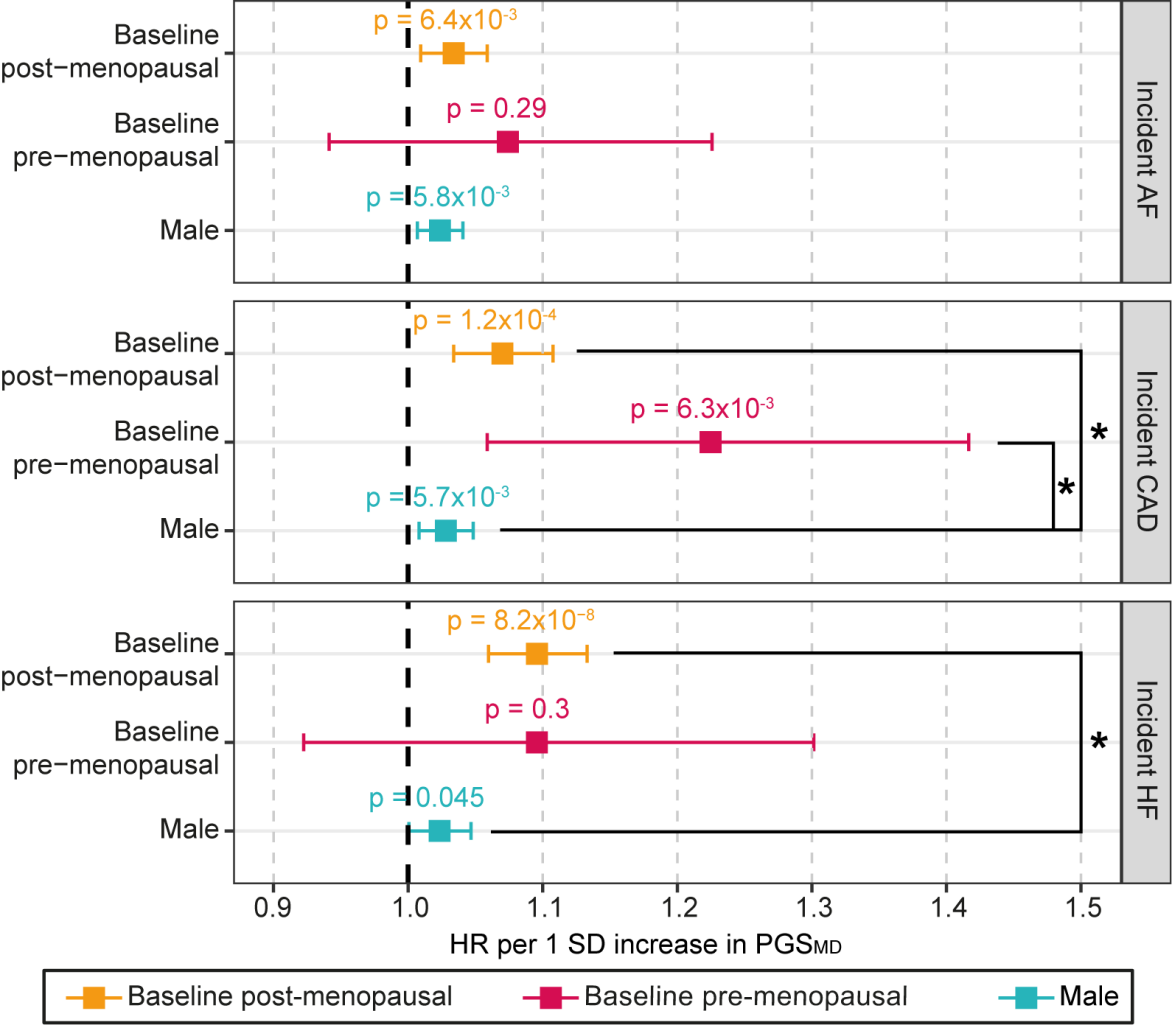
**Change in risk of incident cardiovascular diseases per SD increase in PGS_MD_ in the UK Biobank cohort, stratified by menopausal status.** Associations for females who were baseline postmenopausal (orange) and baseline premenopausal (pink) were estimated with Cox proportional hazards regression models, including body mass index, smoking status, genotyping array, and 20 genetic PCs as covariates. The estimates for the male cohort (blue) are shown for the purpose of comparison. The *X* axis shows the hazard ratio (HR) per SD increase in the polygenic score (PGS), with *P* values labeled, and error bars indicate 95% CIs. *A statistically significant difference (2-sided Wald test *P*<0.05) in the log(HR) values between the corresponding female cohort and male cohort. The dark gray line indicates an HR of 1. AF indicates atrial fibrillation; CAD, coronary artery disease; HF, heart failure; and MD, major depression.

We further explored the association between PGS_MD_ and incident CVDs in females of the UK Biobank stratified by their self-reported menopausal status at baseline (n_post_=114 375 and n_pre_=27 334). As expected from previous evidence on the increase in CVD risks after menopause,^15^ the incidence of CVD was higher in the postmenopausal group (6.0%, 2.9%, and 3.1% for AF, CAD, and HF, respectively) compared with the premenopausal group (0.8%, 0.7%, and 0.5% for AF, CAD, and HF, respectively; χ^2^ *P*<2×10^−^^16^; Table S3). Using a nominal significance (*P*<0.05) threshold, we found that PGS_MD_ was significantly associated with increased incident CAD regardless of baseline menopausal status (HR_CAD-pre_=1.22 [95% CI, 1.06–1.42]; *P*=6.3×10^−^^3^; HR_CAD-post_=1.07 [95% CI, 1.03–1.11]; *P*=1.2×10^−^^4^). The associations with CAD risk in both the baseline premenopausal and postmenopausal female groups were higher than in males (2-sided Wald test *P*=0.019 and 0.047, respectively; Figure 3). A 1-SD increase in PGS_MD_ was also associated with incident AF and HF in the baseline postmenopausal females (HR_AF-post_=1.03 [95% CI, 1.01–1.06]; *P*=6.4×10^−^^3^; HR_HF-post_=1.10 [95% CI, 1.06–1.13]; *P*=8.2×10^−^^8^), where the association with incident HF was significantly higher than the male cohort (2-sided Wald test *P*=8.9×10^−^^4^). Despite risk estimates that were higher than or similar to those observed in the postmenopausal cohort, the associations between PGS_MD_ with incident AF or HF in the baseline premenopausal group were not statistically significant (HR_AF-pre_=1.07 [95% CI, 0.94–1.23]; *P*=0.29; HR_HF-pre_=1.10 [95% CI, 0.92–1.30]; *P*=0.30), which may be a reflection of the much smaller sample size for the premenopausal cohort (Figure 3).

### The Associations Between the Genetic Risk of MD and CVDs Are Partly Mediated by Body Mass Index, Hypercholesterolemia, Hypertension, and Smoking

To gain mechanistic insight into the association between PGS_MD_ and CVD risk, we performed a sex-stratified mediation analysis in the UK Biobank cohort to investigate whether traditional modifiable CVD risk factors could explain the observed sex difference. Although the association between PGS_MD_ and CVD risk in females was found to be partly mediated by many traditional risk factors (namely, body mass index, hypercholesterolemia, hypertension, and smoking), given that the proportion of CVD risk explained by each of the risk factors was higher in males (though not all statistically significant after multiple testing corrections), the observed sex differences in the MD-CVD associations was unlikely to be explained by these traditional risk factors (Table 2). For example, baseline body mass index mediated 34% (95% CI, 21%–78%; *P*=6×10^−^^4^) of the association between PGS_MD_ and incident AF in males but only 25% (95% CI, 18%–41%; *P*<2×10^−^^16^) in females (Table 2).

**Table 2.**
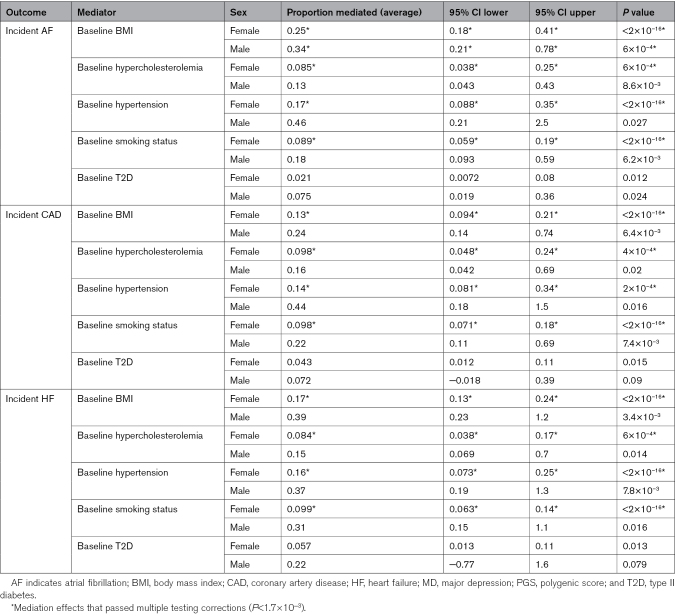
Sex-Stratified Mediation Analysis of Risk Factors Modeled as Mediators of the Association Between PGS_MD_ and Incident Cardiovascular Diseases in the UK Biobank

### Genetic Risk of MD Is Associated With Increased CVD Risk in Females Even in the Absence of Psychiatric Diagnosis or Psychiatric Medication Use

**Figure 4.**
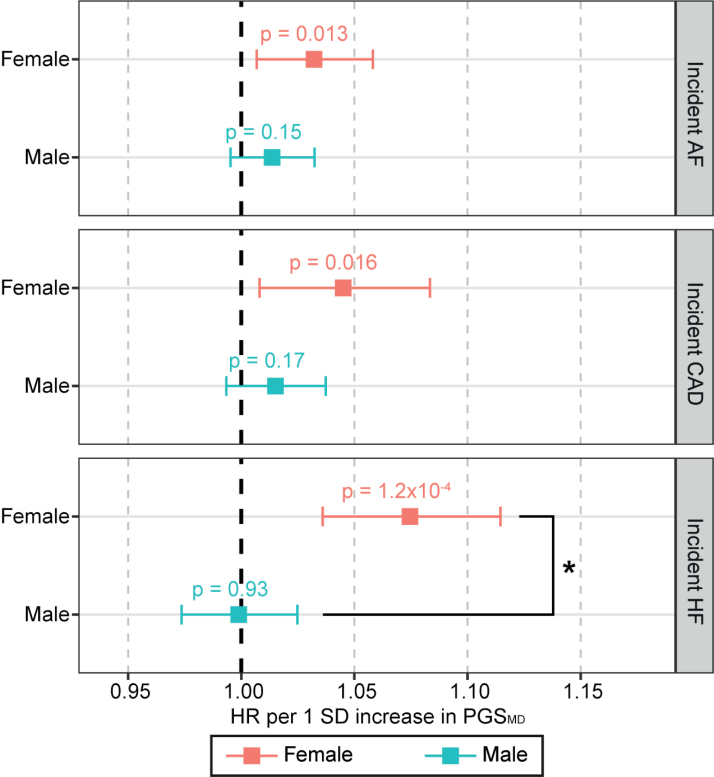
**Change in risk of incident cardiovascular diseases per SD increase in PGS_MD_ among UK Biobank individuals with no diagnosis of psychiatric disorders and psychiatric medications.** Associations for the sex-stratified cohorts (red: female; blue: male) were estimated with Cox proportional hazards regression models, including body mass index, smoking status, genotyping array, and 20 genetic PCs as covariates. The *x* axis shows the hazard ratio (HR) per SD increase in the polygenic score (PGS), with *P* values labeled, and error bars indicate 95% CIs. *A statistically significant difference in the log(HR) values between females and males (2-sided Wald test *P*<0.05). The dark gray line indicates an HR of 1. AF indicates atrial fibrillation; CAD, coronary artery disease; HF, heart failure; and MD, major depression.

To dissociate the effects of behavioral changes or medication use as a consequence of depression diagnosis, sex-stratified Cox proportional hazards regression analysis was performed between PGS_MD_ and incident CVD risks among UK Biobank participants who had no psychiatric disorder diagnosis and were not on any psychiatric medication (n=286 162). Among these individuals, we observed a nominally positive association of PGS_MD_ with incident AF (HR_AF-female_=1.03 [95% CI, 1.01–1.06]; *P*=0.013), incident CAD (HR_CAD-female_=1.05 [95% CI, 1.01–1.08]; *P*=0.016), and incident HF (HR_HF-female_=1.07 [95% CI, 1.04–1.11]; *P*=1.2×10^−^^4^) in females only (Figure 4).

### Replication of Main Findings in the BioVU Cohort

We repeated our analysis in the independent BioVU cohort (n=49 057). Similar to the UK Biobank cohort, after multiple testing corrections (*P*<2.8×10^−^^3^ was used to indicate statistical significance), we observed a positive association of PGS_MD_ with prevalent CAD in females of the BioVU cohort (odds ratio [OR_CAD-female_]=1.08 [95% CI, 1.03–1.12]; *P*=4.3×10^−^^4^) but not in males (OR_CAD-male_=1.04 [95% CI, 1.00–1.08]; *P*=0.044; Figure S2). The difference in risk estimates between the 2 sexes did not reach statistical significance (2-sided Wald test *P*>0.05) possibly due to the smaller sample sizes (Figure S2). The associations between PGS_MD_ and HF risks passed the nominal significance threshold in both sexes (OR_HF-female_=1.07 [95% CI, 1.02–1.11]; *P*=3.6×10^−^^3^; OR_HF-male_=1.06 [95% CI, 1.02–1.10]; *P*=7.2×10^−^^3^) but did not pass multiple testing corrections, likely due to the small sample size. Unlike the UK Biobank, we did not observe a significant association between PGS_MD_ and prevalent AF in either sex. A nominally significant genetic association of PGS_MD_ with HF in both sexes and with CAD in females was observed in the sensitivity analyses adjusting for the diagnosis of psychiatric disorders and antidepressant medication use (Figure S3).

## Discussion

Using data from the UK Biobank cohort, we show for the first time the association between genetic predisposition to MD with incident AF and HF in females but not in males and validate a previously reported sex difference (increased risk in women) in the association with CAD. Mendelian randomization analysis showed evidence to reject a lack of causal association between MD diagnosis and all 3 CVD risks in females, as well as between MD diagnosis and CAD risk in males; however, these results should be interpreted with caution due to the limitations of current Mendelian randomization methods in calculating causal estimates for binary exposures.^18^ As we observed a larger number of CVD cases in males and, therefore, had greater power to detect a risk estimate equivalent to that found in females, the observed sex difference in the depression-CVD associations was unlikely to be driven by differences in statistical power. We also show for the first time that the genetic risk of depression is associated with increased risks of CVDs even in the absence of psychiatric diagnosis or psychiatric medication use, and this association is, thus, not simply a consequence of behavioral changes or medication following a depression diagnosis. Interestingly, the association with incident CAD was consistent among females who were premenopausal at baseline (average age of 44.9 years at baseline), as well as in older females who were postmenopausal at baseline (mean age of 60.8 years at baseline), while the increased risk of incident AF and HF was only observed in the latter group. Our results from mediation analysis suggest that although the depression-CVD link is partly mediated by traditional CVD risk factors, these do not explain the sex difference observed in the UK Biobank. Additional risk factors need to be investigated to understand the driver of the higher CVD risks in women with a higher genetic risk of MD.

We repeated these analyses in the independent BioVU cohort and applied the same multiple testing correction threshold as UK Biobank. After multiple testing corrections, consistent with the higher risk of CAD in females observed in the UK Biobank, we observed a statistically significant association between the genetic risk of MD and increased CAD risk in females but not males in BioVU although this difference in effect between males and females was not statistically significantly different. We also observed a nominally significant association between PGS_MD_ and HF risks in both sexes but did not find any association with AF risks in either sex in BioVU. The lack of replication of the sex differences in BioVU could potentially be explained by lower statistical power, given the much smaller sample size in BioVU (≈49 000 in BioVU versus ≈345 000 in the UK Biobank). Index event bias could also distort the associations between PGS_MD_ and CVDs in BioVU. The Vanderbilt University Medical Center is a tertiary health care center; as such, the BioVU participants were individuals who sought specialized care at the Vanderbilt University Medical Center and, thus, were not representatives of the general population. Potential index event bias in the BioVU data is demonstrated by the negative association between schizophrenia and BD PGSs with CAD and HF risk in males. Though the UK Biobank is not entirely free of index event bias, it is more representative of the general population (not relying on hospital records) than BioVU. Furthermore, analysis in the UK Biobank included only incident CVD cases, while BioVU analysis included all CVD cases due to the nature in which participants were recruited (not prospective recruitment such as UK Biobank). At the same time, the diagnostic strategies of psychiatric disorders are likely to differ between the United Kingdom and the United States.^19^ Independent replication in a large population cohort will provide more robust validation of our findings.

A few observational studies have found depression to be associated with increased AF, CAD, and HF risk in women, and such associations were either weaker or absent in men.^12,13,20^ However, observational studies are prone to unmeasured confounder bias and reverse causation. These studies also have not been able to determine if this risk is independent of psychiatric medication use. Previous genetic analyses that utilize GWAS summary data to assess causality have shown that an increased genetic risk for MD is associated with an increased CAD and HF risk.^16^ However, these studies lacked a sex perspective, except for a previously reported genetic association between MD PGS and CAD observed in females but not males in a (smaller) BioVU cohort.^17^

In this study, we also observed comparable risk estimates between PGS_MD_ and incident CVDs among 2 cohorts of women who were at different menopausal stages at enrollment into the UK Biobank although the risk estimates in the premenopausal cohort were restricted by sample size and, thus, did not reach statistical significance for AF and HF. The menopause transition involves extensive changes in sex hormones, body composition, and lipid profiles, which can increase CVD risk in women postmenopause.^15^ Our findings demonstrate that depression may be an important consideration in CVD risk assessment regardless of menopausal stage.

The MD GWAS data from which our PGS_MD_ were derived identified loci known to be associated with body mass index,^21^ which corroborates our finding that the association between PGS_MD_ and CVD risks was partly mediated by body mass index. However, sex-specific mediation analyses suggest that the more pronounced CVD risk associated with higher genetic predisposition to MD in females versus males is not explained by traditional CVD risk factors. We note that these mediation analyses were likely to be sensitive to confounders, which could lead to a violation of the assumption of mediation analysis. However, this assumption is often difficult to test, particularly in our study, due to the complex relationships among the traditional CVD risk factors. Our findings are intended to highlight the importance of further investigation into the mechanisms that mediate the association between the genetic risk of MD and CVD risks, particularly mechanisms underlying the sex difference. Previous studies have suggested hormonal dysregulation and proinflammatory responses as 2 potential mechanisms underlying this female preponderance.^22^ Specific subtypes of depression, such as postpartum depression and postmenopausal depression, suggest the involvement of hormone fluctuations in depression among females.^22^ Interestingly, Takotsubo cardiomyopathy, often triggered by emotional or physical stress, is more common in postmenopausal women (90% of cases).^23^ At the same time, elevated inflammatory biomarkers, such as interleukin-6 and tumor necrosis factor alpha, are reportedly higher in depressed women relative to depressed men, suggesting a sex-differential inflammatory response to MD.^22^ These proinflammatory cytokines are linked to CVD risks.^24^ Furthermore, a previous sex-stratified GWAS of depression highlighted gene expression regulation as a significantly enriched biological process in both females and males though the regulatory genes involved appeared to be sex-specific.^25^ At the same time, tissues involved in cardiovascular homeostasis are found to show abundant sex differences in gene expression.^26^ The evidence above suggests differential transcriptomic regulation as a potential mechanism underlying the sex-specific MD-CVD associations. In addition to biological mechanisms, many social factors associated with stress, such as long hours of caregiving, affect the risks of CVD more in women than men. These social factors may also contribute to the stronger MD-CVD link in women.^27^ Further studies, taking into consideration the different depression subtypes and symptoms, are required to understand the role of these factors in the heightened risk of CVDs among females with depression.

Currently, the QRESEARCH Cardiovascular Risk Algorithm version 3 (QRISK3) (UK) is the only CVD risk prediction calculator that incorporates diagnoses of severe mental illnesses as risk factors for primary prevention.^28^ In New Zealand, individuals with severe mental illness are advised to undergo earlier and more frequent risk assessments.^29^ In Australia, severe mental illnesses are recommended as a reclassification factor and to be used to refine CVD risk categorization for individuals whose risk predicted from traditional risk factors lies close to a threshold of a higher risk group.^30^ Furthermore, the American Heart Association has recommended incorporation of a depression diagnosis as a risk factor of adverse prognosis among patients with acute coronary syndrome,^31^ but the diagnosis of depression is not currently incorporated into the calculation of CVD risk (pooled cohort equations) in the United States.^32^ Given the observation of higher CVD risk in individuals who are genetically predisposed to depression, even in the absence of a depression diagnosis, future studies investigating any benefit of including such information in risk tools are warranted, especially in women.

The strengths of our study lie, first, in the investigation of sex-specific psychiatric-CVD comorbidity, and the ability to dissociate the effects of depression from medications and behavior changes following diagnosis. However, the limitations of our study need to be acknowledged. We derived PGSs from sex-combined summary statistics of GWAS, which might fail to capture genetic variants that confer sex-differential associations. The between-sex genetic correlations for BD, MD, and schizophrenia are reported to range between 0.86 and 1, indicating moderate to subtle sex differences in the genetic architecture of these psychiatric disorders.^33^ Nevertheless, PGSs derived from sufficiently powered sex-stratified GWAS studies, which are becoming increasingly available for neuropsychiatric and cardiovascular traits,^33,34^ will provide additional information on the sex-differential genetic factors in diseases. In addition, we defined disease status using self-reported answers to questionnaires, electronic health records (*International Classification of Diseases*, *Ninth Edition*; *International Classification of Diseases*, *Tenth Edition*; and OPCS4), and death records. As self-reported data and electronic health records may be prone to ascertainment bias and missingness, manual adjudication of disease status by qualified clinicians will provide more reliable phenotype definitions. However, cohorts with genetic data and phenotype definitions of such granularity are rare and often lack sufficient sample sizes; therefore, in this study, we utilized multiple sources for phenotype definitions (including *International Classification of Diseases*, *Ninth Edition*; *International Classification of Diseases*, *Tenth Edition*; OPCS4 operation; and death records) to minimize the bias potentially introduced by misclassification and measurement error. Moreover, due to the lower number of CVD cases among females in the UK Biobank, our analysis in the female cohort had lower power. In addition, the analysis of CVD risks stratified by menopausal states did not account for the potential effects of any menopause treatments, which are known to impact CVD risks.^35^ Over 47.5% (54 350 of 114 375) of baseline postmenopausal females in this study self-reported having used hormone replacement therapy at baseline (versus only 1.5% of the baseline premenopausal cohort). This is an important consideration for future studies to dissociate the effects of such treatments on the CVD risk among individuals with a genetic predisposition to MD. Furthermore, the healthy-volunteer bias in the UK Biobank is well documented, where individuals with severe mental illnesses are underrepresented compared with the general population.^36^ While an underrepresentation of depression is also present in this cohort, it is estimated that this bias is likely more prominent for more severe mental illnesses, namely, schizophrenia.^36^ Finally, due to the sparsity of data in diverse ancestry populations, our analysis was restricted to individuals with genetically inferred European ancestry. Addressing this caveat requires sufficiently powered GWAS of depression and biobank-style data in diverse groups, which will become increasingly available in the future. Given large racial/ethnic disparities in CVD outcomes in countries such as the United States, United Kingdom, and Australia, due to disparities in socioeconomic status and health care access, it is imperative for future studies aimed at developing improved CVD prevention strategies to consider these factors to ensure more equitable CVD prevention.

To our best knowledge, this is the first study that has explored a sex-specific association between the genetic risk of 3 different psychiatric disorders and risks of AF, CAD, and HF, with further stratification for menopause status. CVD risk calculators that do not incorporate psychiatric disorders as a predictor are reported to underestimate CVD risk by 30% and 60% in men and women, respectively.^37^ Our findings highlight the need for studies focused on understanding sex-specific drivers of CVD risk in the presence of depression to inform the development of risk predictors and prevention strategies in the context of comorbidity. Furthermore, our findings underscore the importance of effective implementation of CVD screening in females with a predisposition to or diagnosis of depression. Several studies have documented gender disparities in the use of cardiovascular tests, including assessment of CVD risk.^38^ This may be especially relevant among young women, who show a high depression prevalence but are traditionally perceived to have lower CVD risk.

## ARTICLE INFORMATION

### Acknowledgments

The authors would like to thank the research participants and employees of 23andMe, Inc, for making this work possible. The full genome-wide association study summary statistics for the 23andMe discovery data set will be made available through 23andMe to qualified researchers under an agreement with 23andMe that protects the privacy of the 23andMe participants. Please visit https://research.23andme.com/collaborate/#dataset-access/ for more information and to apply to access the data. The data set(s) used for the BioVU analyses described were obtained from Vanderbilt University Medical Center’s BioVU that is supported by numerous sources: institutional funding, private agencies, and federal grants. These include the National Institutes of Health (NIH)–funded Shared Instrumentation Grant S10RR025141 and Clinical and Translational Science Awards (CTSA) grants UL1TR002243, UL1TR000445, and UL1RR024975. Genomic data are also supported by investigator-led projects that include U01HG004798, R01NS032830, RC2GM092618, P50GM115305, U01HG006378, U19HL065962, and R01HD074711; additional funding sources are listed at https://victr.vumc.org/biovu-funding/. Analysis of the BioVU dataset in this publication was supported by grant R01 HG011405, partially funded by the Office of Research on Women’s Health, the Office of the Director, NIH, and the National Human Genome Research Institute. Its contents are solely the responsibility of the authors and do not necessarily represent the official views of the Office of Research on Women’s Health or the National Human Genome Research Institute.

### Sources of Funding

Dr Jiang is supported by a National Health and Medical Research Council (NHMRC) IDEAS grant 2000637. Dr Singh is supported by the American Heart Association Predoctoral Fellowship (grant AHA827137). Dr Davis is supported by funding from the National Institute of Mental Health (grant R56MH120736), the National Human Genome Research Institute, and the Office of Research on Women’s Health (grant R01HG011405). Dr Wray is supported by an NHMRC Program Grant (1113400) and an NHMRC Investigator Grant (1173790). Dr Shah is supported by funding from the NHMRC Program Grant (1113400), the NHMRC Early Career Fellowship (grant APP1142495), and the National Heart Foundation Future Leader Fellowship (grant 105638).

### Disclosures

None.

### Supplemental Material

Supplemental Methods

Tables S1–S10

Figures S1–S4

References 39–62

## Supplementary Material


