# Supplementary material for: Sex-Specific Association Between Genetic Risk of Psychiatric Disorders and Cardiovascular Diseases
Source: Circ Genom Precis Med. 2024 Nov 29;17(6):e004685. doi: 10.1161/CIRCGEN.124.004685 (PMC11651350; doi:10.1161/CIRCGEN.124.004685)
Supplement: Supplementary file 1 [file hcg-17-e004685-s001.pdf]

SUPPLEMENTAL MATERIAL

Supplemental Methods

UK Biobank cohort summary

The UK Biobank cohort consists of over 500,000 participants (aged 40-69 years at baseline) from the UK, who were recruited between 2006 and 2010 [39]. Diverse phenotypic and genomic data were collected from participants, such as clinical diagnosis, physical and biochemical measurements, and answers from questionnaires. Consent was collected from all participants. The current study was conducted using the UK Biobank Resource under Application Number 12505. This research is covered by The University of Queensland Human Research Ethics Committee approval (HREC number 2020/HE002938).

Using information provided by the UK Biobank genotype data release, quality control was performed to remove individuals who were outliers for heterozygosity and genotype missing rates, who were excluded from kinship inference, who showed evidence of putative sex chromosome aneuploidy, and who showed mismatching self-reported and genetically inferred sex (definitions of exclusion criteria are described in Bycroft et al. [39]). Individuals who have withdrawn from the study or have missing phenotypes [BMI or smoking status] were excluded from the analysis. After quality control, a total of 345,169 unrelated (genetic relatedness < 0.05) individuals of European ancestry (inferred from the genetic data) were subjected to analysis in this study (ancestry calling is described in Yengo et al. [40]).

BioVU cohort summary

VUMC is a tertiary care centre that provides inpatient and outpatient care in middle Tennessee, US. The VUMC EHR system, established in 1990, includes data on billing codes from the

International Classification of Diseases 9th and 10th editions (ICD-9 and ICD-10), Current Procedural Terminology (CPT) codes, laboratory values, reports, and clinical documentation. In 2007, VUMC launched a biobank, BioVU, which links a patient's DNA sample to their EHR. The BioVU consent form is provided to patients in the outpatient clinic environments at VUMC. The VUMC Institutional Review Board oversees BioVU and approved this project (IRB#172020). At VUMC, all medical record data were extracted from the EHRs of 72,634 individuals of European ancestry, determined by genetic ancestry analysis. To reduce the missingness of clinical data and enrich the sample for patients who receive their primary care at VUMC, our analysis included only individuals who met the "medical home" definition, requiring the presence of any five codes on different days over a period of at least three years. We further excluded individuals who did not have a body mass index (BMI) measurement or age recorded in the EHRs, leaving a total of 49,057 individuals for analysis.

CVD and risk factor phenotype ascertainment in UK Biobank

UK Biobank phenotypic data collected between 2006 and March 2023 was used for phenotype extraction. Detailed inclusion and exclusion criteria of all disease phenotypes in the UK Biobank are provided in Supplemental Table 4 and Supplemental Table 5.

AF cases were defined by the presence of self-reported atrial fibrillation or atrial flutter, self-reported cardioversion operation, ICD-10 (I48, I48.0-4, I48.9) or ICD-9 (4273) billing codes indicative of atrial fibrillation or atrial flutter, OPCS4 procedures (K57.1, K62.1-4), or death causes indicative of atrial fibrillation or atrial flutter (same as the ICD-10 codes).

CAD cases were ascertained based on myocardial infarction or coronary revascularisation. Myocardial infarction was defined by self-reported heart attack/myocardial infarction, or ICD-10

(I21.X, I22.X, I23.X, I24.1, I25.2) or ICD-9 (4109, 4119, 4129) billing codes for myocardial infarction. Coronary revascularisation was defined by OPCS4 codes for coronary artery bypass grafting (K40.1–4, K41.1–4, or K45.1–5) or coronary angioplasty with or without stenting (K49.1–2, K49.8–9, K50.2, K75.1–4, K75.8–9).

HF cases were ascertained by the presence of self-reported heart failure/pulmonary odema or cardiomyopathy, ICD-10 (I11.0, I13.0, I13.2, I25.5, I42.0, I42.5, I42.8, I42.9, I50.0, I50.1, I50.9) or ICD-9 (4254, 4280, 4281, 4289) billing codes indicative of heart/ventricular failure or cardiomyopathy, or death causes indicative of heart/ventricular failure or cardiomyopathy. Individuals with hypertrophic cardiomyopathy, defined by self-reported illness, ICD-10 (I42.1, I42.2) billing codes, ICD-9 (4251) billing codes or death causes, were excluded from the HF analyses.

Hypercholesterolaemia cases were defined by self-reported high cholesterol, ICD-10 (E780) or ICD-9 (27200, 27209) codes for hypercholesterolaemia, or death causes indicative of hypercholesterolaemia. Individuals who were not defined as a hypercholesterolaemia case, but self-reported to be on cholesterol-lowering medication were removed from the hypercholesterolaemia controls.

Hypertension cases were defined by self-reported hypertension, ICD-10 (I10) or ICD-9 (4010, 4011, 4019) codes for essential (primary) hypertension, or death causes indicative of hypertension. Individuals who were not defined as a hypertension case but self-reported to be taking blood pressure medications were removed from the hypertension controls.

Type II diabetes (T2D) cases were defined by self-reported T2D, ICD-10 (E11.X) or ICD-9 (25000, 25010) codes for non-insulin-dependent diabetes mellitus, or death causes indicative of

T2D. Individuals who were not defined as a hypertension case but had Type I diabetes or were taking insulin were removed from the T2D controls. Type I diabetes cases were defined by self-reported Type I diabetes, ICD-10 (E10.X) codes, ICD-9 (25001, 25011) codes, or death causes.

For each CVD, prevalent cases consisted of individuals who had disease-associated phenotypes recorded at baseline or after recruitment into UK Biobank, defined using self-reported illnesses, self-reported operation codes, operative procedures (OPCS4), ICD-9 diagnoses, ICD-10 diagnoses and primary or contributory death causes (not all criteria were used for all phenotype definitions). Incident cases were defined as individuals who did not self-report any disease-associated illness or operation code at initial assessment (determined using "Date of attending assessment centre", "Interpolated Year when non-cancer illness first diagnosed" and "Interpolated Year when operation took place"), and did not have any disease-associated OPCS4 procedure or diagnosis (ICD-9 or ICD-10) prior to or at the time of initial assessment (determined using "Date of first operative procedure - OPCS4", "Date of first in-patient diagnosis - ICD-9" and "Date of first in-patient diagnosis - ICD-10"). For prevalent cases who answered "Date uncertain or unknown" or "Preferred not to answer" to the self-reported illness diagnosis or operation date, these dates were set to be the date on which they attended the assessment centre when they self-reported the illness or operation. The process of defining prevalent and incident cases is presented in Figure 1.

For the mediation analysis, the baseline status of the disease risk factors of CVDs (hypercholesterolaemia, hypertension, and T2D) was defined using criteria in Supplemental Table 4. Incident cases of these risk factors, defined as described above, were removed from the mediation analysis.

We retrieved participant information on BMI and smoking status (whether a person has smoked ever, defined as current or previous smokers) at baseline. The ages at baseline (in years) of individuals were calculated using "Date of attending assessment centre" and "Year of birth". Summary of characteristics for the whole cohort and the cohort with no psychiatric diagnosis and medications are shown in Table 1 and Supplemental Table 6 respectively.

Psychiatric phenotype ascertainment in UK Biobank

BD cases were ascertained by the presence of ICD-10 (F30.0, F30.1, F30.2, F30.8, F30.9, F31.X) or ICD-9 (2960, 2961, 2969) codes for manic episode or bipolar affective disorder. SCZ cases were defined using ICD-10 (F20.X, F21.X, F22.X, F23.X, F24.X, F25.X, F28.X, F29.X) or ICD-9 (2953, 2959) codes for schizophrenia, schizotypal and delusional disorders. MD cases were defined by ICD-10 (F32.X, F33.X, F34.X, F38.X, F39.X) or ICD-9 (3119, 2962) codes for depressive episode, recurrent depressive disorder, persistent mood (affective) disorders, other mood (affective) disorders or unknown mood (affective) disorders. Individuals were removed from the MD cases if they were a case for BD or SCZ, had self-reported schizophrenia (1289), had an ICD-10 code of multiple personality disorder (F44.8), or were taking antipsychotics. The controls for BD, SCZ and MD were individuals who had no diagnosis of psychiatric disorders and were not taking any psychiatric medication.

BioVU phenotype ascertainment

For the BioVU cohort, we defined phenotypes based on Phecodes, which are higher-order combinations of at least two related ICD codes, occurring on two different days, using the R PheWAS package [41] (detailed Phecodes are shown in Supplemental Table 7). Smoking status was ascertained by "tobacco use disorder" (Phecode 318). The median BMI and median age of

individuals were selected from assessments taken at chronological visits, available in the medical records at VUMC. Summary of characteristics is presented in Supplemental Table 1.

GWAS summary statistics

Association summary statistics derived from European-ancestry or predominantly European-ancestry cohorts were retrieved for three CVDs (AF, CAD and HF) and three psychiatric disorders (BD, MD and SCZ), and were used for generating PGS in the UK Biobank and BioVU cohorts (datasets summarised Supplemental Table 8). To avoid discovery bias, GWAS summary statistics used for single nucleotide polymorphism (SNP) selection and per-allele weight estimation were derived from studies that did not include individuals from the corresponding target cohorts (UK Biobank or BioVU).

Polygenic score generation

PGS is an estimate of the lifetime genetic liability of an individual to a trait. We define a PGS of an individual, j, as a weighted sum of SNP allele counts: $\sum_{i=1}^{m} \hat{b}_i x_{ij}$, where m is the number of SNPs included in the predictor, $\hat{b}_i$ is the per-allele weight (based on the estimated effect of the allele on trait value) for the SNP, x_{ij} is a count of the number (0, 1, or 2) of trait-associated alleles of SNP i in individual j.

Several methods are available for optimising the choice of SNPs and the per-allele weights for PGS calculation. For analysis in the UK Biobank cohort, we used SBayesR [42] which implements a Bayesian approach to model genetic architecture, is computationally efficient, and does not require a training dataset for identifying trait-associated SNPs and estimating per-allele weights [42,43]. A linkage disequilibrium (LD) reference dataset was downloaded from the SBayesR website

(banded LD matrix) and default settings were used unless otherwise stated (--exclude-mhc; --chain-length 50000; --burn-in 10000; --no-mcmc-bin) [42]. For each disease, we applied PLINK1.9 [44] to calculate the PGSs (based on SBayesR SNPs and weights) in UK Biobank individuals. For BioVU, PGSs were generated using Polygenic Risk Score–Continuous Shrinkage (PRS-CS) (auto) [45]. The LD reference panel was constructed from 503 European samples in the 1000 Genomes Project Phase 3.41.

The PGS distribution was scaled to a mean of zero and standard deviation (SD) of 1; therefore, effect size estimates in subsequent regression analyses were interpreted as changes in outcome per 1-SD increase in PGS. We generated PGS for 345,169 unrelated, European-ancestry individuals [186,683 (54.1%) females and 158,486 (45.9%) males] from the UK Biobank, and 49,057 individuals [28,094 (57.3%) females and 20,963 (42.7%) males] from BioVU. To validate the PGS, logistic regression was used to examine the association with disease prevalent cases in the prediction cohort (Supplemental Table 9), including sex, age, genotyping array (UK Biobank only), and genetic principal components (PC) (20 PCs for UK Biobank and 10 PCs for BioVU) as covariates. PGS performance was evaluated using the area under the receiver operator characteristic curve (AUC) [R package pROC [46] (version 1.18.0)]. The AUC can be interpreted as a probability of a case having a higher PGS than a control. Our models demonstrate comparable AUC values to the corresponding PGSs generated previously using different methods [47-50] (Supplemental Table 9). In the BioVU cohort, we also performed sensitivity analysis by generating PGSs using SBayesR. We confirmed that the PGSs generated by PRS-CS-auto and SBayesR showed a high consistency, supported by Pearson's correlations ranging between 0.78 and 0.89 (Supplemental Figure 4) and comparable prediction accuracy (Supplemental Table 10).

Cox proportional hazards regression analysis

In UK Biobank, for each psychiatric-CVD disorder pair, we performed age-as-time-scale Cox proportional hazards regression to investigate the sex-specific association between psychiatric disorder PGS and incident CVD. For each CVD incident case, we defined their time-to-event as the number of months since birth (estimated using "Year of birth" and "Month of birth"), to the age they were diagnosed with CVD (estimated using "Date of first in-patient diagnosis"), first self-reported CVD illness (estimated using "Interpolated Year when non-cancer illness first diagnosed"), self-reported operation codes (estimated using "Interpolated Year when operation took place"), had the CVD-associated OPCS4 procedures (estimated using "Date of first operative procedure - OPCS4") or died of CVDs (estimated using "Date of death"). If an individual met multiple disease-associated criteria (such as having had both a disease-associated ICD-10 diagnosis and OPCS4 procedure), their time-to-event was defined as the earliest time at which they met any disease-associated criteria. We censored CVD controls at the age of death, age of losing contact (estimated using "Date lost to follow-up"), or age at censoring (March 2023).

The Cox models included BMI, smoking status, genotyping array and 20 genetic PCs as covariates, and were analysed using the "survival" package (version 3.2.11) in R [51]. For the primary analysis in the whole UK Biobank cohort, a $p<2.8\times10^{-3}$ was chosen to indicate statistical significance (multiple testing correction for 18 tests – association of three psychiatric PGS with three CVDs in two sexes). The regression coefficients [log(HR)] for the PGS were compared between sexes using a Wald test (statistical significance declared at two-sided $p<0.05$). Given previously reported genetic correlations between CVD and depression [52], to assess if the PGS_{MD} were associated with CVD risks independently of the CVD PGS, we performed a sensitivity analysis where the PGS for the relevant CVD was fitted as an additional covariate. Due to the bidirectional relationships amongst the different CVDs, we performed a further sensitivity analysis where the PGSs for all

CVDs were fitted as additional covariates. For all subsequent subgroup analyses, which were performed to investigate the consistency in risk estimates, a nominal significance threshold ($p<0.05$) was applied due to the reduction in sample sizes.

Sex-specific MR analysis

While MR can provide insights into causal associations, current MR methods are limited in calculating the causal estimates for binary exposures [18]. Therefore, using the PGS_{MD} as the genetic instrument, we performed sex-specific MR in the UK Biobank to only test the null association between MD diagnosis (prevalent MD) and prevalent CVDs. MR was performed through two-stage least-squares instrumental variable regressions, using the "ivreg" function from the AER R package (version 1.2.10) [53]. The model included age at baseline, genotyping array, BMI, smoking and 20 PCs as covariates. We only reported the direction of effects and p-values of the MR analysis instead of the test statistics, to avoid overextrapolation of the magnitude of causal effect estimate. A $p<8.3\times10^{-3}$ was chosen to indicate statistical significance (multiple testing correction for 6 tests – association of PGS_{MD} with three CVDs in two sexes).

Cox proportional hazards analysis in females stratified by baseline menopausal status

We sought to determine if the association between the genetic risk of MD and risk of incident CVD differed between two groups of women at different stages of menopause at baseline. In the UK Biobank cohort, the menopause status at baseline was determined using answers to the question of "Had menopause?", where females were identified to be post-menopausal at baseline if they answered "yes" to the survey question "Had menopause?" at the initial assessment visit. The mean age of natural menopause is 50 years (interquartile range = 48.0–53.0 years) [15], we thus also conducted analysis in a cohort consisting of females who at baseline were less than 50 years

of age and pre-menopausal (answered “no” to “Had menopause?” at the initial assessment), and either answered “no” or did not answer the “Had menopause” question in subsequent assessments (about 18.7% of females who answered “no” to “Had menopause?” at the initial assessment completed at least one follow-up assessment on menopausal status). Females who at baseline did not answer, answered “preferred not to answer”, or answered “not sure” to the menopause question were excluded from both menopausal groups, while those who in any follow-up assessments reported to have had menopause, answered “preferred not to answer”, or answered “not sure” were also excluded from the baseline pre-menopausal group. A total of 141,709 females in the study met the criteria for either menopausal group, including 114,375 (80.7%) females who self-reported to have had menopause at baseline, and 27,334 (19.3%) females in the baseline pre-menopausal group. We performed Cox proportional hazards regression analysis (as described above) between PGS_{MD} and incident CVDs in the two menopausal groups. BMI, smoking status, genotyping array, and 20 genetic PCs were included in the model as covariates. For each CVD, we applied a Wald test to compare the Cox regression coefficient (beta) for PGS_{MD} in each menopausal group against the previously estimated coefficient in males.

Mediation analysis with CVD-associated risk factors

To explore whether the association between the genetic risk of MD and incident CVDs can be explained by a CVD-associated risk factor, sex-stratified mediation analysis was performed in the UK Biobank cohort using the “mediation” R package (version 4.5.0) [54]. Five risk factors (baseline BMI, hypercholesterolaemia status at baseline, hypertension status at baseline, smoking status at baseline and T2D status at baseline) were modelled as mediators between PGS_{MD} (exposure) and incident CVD (outcome). The mediation models included baseline BMI (except in the analysis of baseline BMI as the mediator), baseline smoking status (except in the analysis of smoking status

as the mediator), age at baseline, genotyping array and 20 genetic PCs as covariates. Each mediation analysis was performed with 10,000 bootstrap simulations to estimate the variance and significance of the mediation, and statistical significance was defined by $p<1.7\times10^{-3}$ (multiple testing correction for three CVDs, five risk factors and two sexes).

Association between the genetic risk of MD and CVD in individuals with no diagnosis of psychiatric disorders or psychiatric medication use

To dissociate the effects of behavioural changes or medication use as a consequence of depression diagnosis, sex-stratified Cox proportional hazards regression analysis was performed between PGS_{MD} and incident CVD risks amongst UK Biobank participants who did not meet the criteria for the three psychiatric disorders, had no ICD diagnosis of personality or neurotic disorders, had no self-reported history of mood disorders, and were not on any antidepressants or antipsychotic medications (detailed phenotype information in Supplemental Table 5). A total of 286,162 individuals from the UK Biobank cohort met the above criteria and were included for analysis.

Logistic regression in BioVU

Findings from UK Biobank were tested for association in the independent BioVU cohort at VUMC. Based on methods used in previous BioVU studies [17], logistic regression was used to estimate the sex-stratified association of psychiatric disorder PGS with prevalent CVDs, including median age, median BMI, smoking status and 10 genetic PCs as covariates. Similar to UK Biobank, statistical significance was declared at $p<2.8\times10^{-3}$ after multiple test correction. In a sensitivity analysis, we additionally adjusted for the presence of a Phecode for any of the psychiatric conditions (BD, MD, SCZ, personality disorders, mood disorders, or anxiety, dissociative and somatoform disorders) (Supplemental Table 7), along with the presence of

antidepressant use, defined as whether a person has ever used an antidepressant. For the sensitivity analysis, as was done for UK Biobank, statistical significance was declared at $p<0.05$.

Supplemental Tables

Supplemental Table 1. Summary of characteristics of the BioVU cohort

		Female		Male	
	Total Number	28094		20963	
	Median age in years (SD)	51.00 (20.80)		56.00 (22.50)	
	Median BMI (SD)	26.76 (7.55)		27.34 (6.60)	
	Tobacco Use Disorder (%)	3129 (11.14)		3486 (16.63)	
	Antidepressant use (%)	16295 (58)		9063 (43.23)	
	Psychiatric conditions (%)	8993 (32.01)		5043 (24.06)	
		Case	Control	Case	Control
AF	N (%)	2256 (8.03)	25838 (91.97)	3416 (16.30)	17547 (83.70)
	Median age (SD)	72.00 (12.62)	49.00 (20392)	69.00 (12.33)	53.00 (22.6)
CAD	N (%)	2881 (10.25)	25213 (97.1)	5487 (26.17)	15476 (73.83)
	Median age (SD)	68 (12.23)	49.00 (20.58)	68.00 (10.95)	50.00(22.84)
HF	N (%)	2514 (8.95)	25580 (91.05)	3183 (15.18)	17,780 (84.82)
	Median age (SD)	67.00 (18.03)	50.00 (20.53)	67.00 (16.65)	54.0 (22.61)

Supplemental Table 2. Direction of effects and p-values from sex-specific MR analysis

Exposure	Outcome	Sex	Direction of effects	p-value
Prevalent MD	Prevalent AF	Female	Positive	2.7×10^{-6}
		Male	Positive	0.014
	Prevalent CAD	Female	Positive	3.6×10^{-8}
		Male	Positive	2.3×10^{-5}
	Prevalent HF	Female	Positive	2.9×10^{-11}
		Male	Positive	0.10

Supplemental Table 3. CVD incidence and average age at baseline of females who were at baseline pre- and post-menopausal in the UK Biobank (*** indicates a chi square $p<2\times10^{-16}$ for comparison of CVD incidence between the two menopausal groups)

		Baseline pre-menopause		Baseline post-menopause		
		Case	Control	Case	Control	
AF	Count (%)	222 (0.8)	27076 (99.1)	6849 (6.0)	106288 (92.9)	***
	Average age at baseline in years (SD)	45.3 (2.5)	44.9 (2.6)	63.6 (4.6)	60.6 (5.4)	
CAD	Count (%)	185 (0.7)	27103 (99.2)	3288 (2.9)	109621 (95.8)	***
	Average age at baseline in years (SD)	45.4 (2.4)	44.9 (2.6)	62.9 (4.9)	60.7 (5.4)	
HF	Count (%)	130 (0.5)	27181 (99.4)	3510 (3.1)	110383 (96.5)	***
	Average age at baseline in years (SD)	45.3 (2.4)	44.9 (2.6)	63.7 (4.8)	60.7 (5.4)	

Supplemental Table 4. UK Biobank CVD and associated risk factors phenotype ascertainment criteria

Disease	Criteria	Data field	Data field description	Codes
AF	Inclusion	20002	Non-cancer illness code, self-reported	1471, 1483
		20004	Operation code	1524
		41270	ICD-10	I48, I480, I481, I482, I483, I484, I489
		41271	ICD-9	4273
		41272	Operative procedures - OPCS4	K571, K621, K622, K623, K624
		40001 and 40002	Primary and secondary causes of death	I48, I480, I481, I482, I483, I484, I489
CAD	Inclusion	20002	Non-cancer illness code, self-reported	1075
		41270	ICD-10	I210, I211, I212, I213, I214, I219, I220, I221, I228, I229, I230, I231, I232, I233, I235, I236, I238, I241, I252
		41271	ICD-9	4109, 4119, 4129
		41272	Operative procedures - OPCS4	K401, K402, K403, K404, K411, K412, K413, K414, K451, K452, K453, K454, K455, K491, K492, K498, K499, K502, K751, K752, K753, K754, K758, K759
		40001 and 40002	Primary and secondary causes of death	I210, I211, I212, I213, I214, I219, I220, I221, I228, I229, I230, I231, I232, I233, I235, I236, I238, I241, I252
HF	Inclusion	20002	Non-cancer illness code, self-reported	1076, 1079
		41270	ICD-10	I110, I130, I132, I255, I420, I425, I428, I429, I500, I501, I509
		41271	ICD-9	4254, 4280, 4281, 4289
		40001 and 40002	Primary and secondary causes of death	I110, I130, I132, I255, I420, I425, I428, I429, I500, I501, I509
	Exclusion	20002	Non-cancer illness code, self-reported	1588
		41270	ICD-10	I421, I422
		41271	ICD-9	4251
		40001 and 40002	Primary and secondary causes of death	I421, I422
Hyper-cholesterolemia	Inclusion for cases	20002	Non-cancer illness code, self-reported	1473
		41270	ICD-10	E780
		41271	ICD-9	27200, 27209
		40001 and 40002	Primary and secondary causes of death	E780
	Exclusion from controls	6177	Medication for cholesterol, blood pressure or diabetes	1 (Cholesterol lowering medication)
		6153	Medication for cholesterol, blood pressure, diabetes, or take exogenous hormones	1 (Cholesterol lowering medication)
Hypertension	Inclusion for cases	20002	Non-cancer illness code, self-reported	1065, 1072
		41270	ICD-10	I10
		41271	ICD-9	4010, 4011, 4019
		40001 and 40002	Primary and secondary causes of death	I10
	Exclusion from controls	6177	Medication for cholesterol, blood pressure or diabetes	2 (Blood pressure medication)
		6153	Medication for cholesterol, blood pressure, diabetes, or take exogenous hormones	2 (Blood pressure medication)
T2D	Inclusion for cases	20002	Non-cancer illness code, self-reported	1223
		41270	ICD-10	E110, E111, E112, E113, E114, E115, E116, E117, E118, E119
		41271	ICD-9	25000, 25010
		40001 and 40002	Primary and secondary causes of death	E110, E111, E112, E113, E114, E115, E116, E117, E118, E119
	Exclusion from controls	20002	Non-cancer illness code, self-reported	1222
		41270	ICD-10	E100, E101, E102, E103, E104, E105, E106, E107, E108, E109
		41271	ICD-9	25001, 25011
		6177	Medication for cholesterol, blood pressure or diabetes	3 (Insulin)
		6153	Medication for cholesterol, blood pressure, diabetes, or take exogenous hormones	3 (Insulin)
		40001 and 40002	Primary and secondary causes of death	E100, E101, E102, E103, E104, E105, E106, E107, E108, E109

Supplemental Table 5. UK Biobank psychiatric disorder phenotype ascertainment criteria

Disease	Criteria	Data field	Data field description	Codes
BD	Inclusion	41270	ICD-10	F300, F301, F302, F308, F309, F310, F311, F312, F313, F314, F315, F316, F317, F318, F319
		41271	ICD-9	2960, 2961, 2969
SCZ	Inclusion	41270	ICD-10	F200, F201, F202, F203, F204, F205, F206, F208, F209, F21, F220, F228, F229, F230, F231, F232, F233, F238, F239, F24, F250, F251, F252, F258, F259, F28, F29
		41271	ICD-9	2953, 2959
MD	Inclusion	41270	ICD-10	F320, F321, F322, F323, F328, F329, F330, F331, F332, F333, F334, F338, F339, F340, F341, F348, F349, F380, F381, F388, F39
		41271	ICD-9	3119, 2962
	Exclusion	BD cases		
		SCZ cases		
		41270	ICD-10	F448
		20002	Non-cancer illness code, self-reported	1289
		20003	Treatment/medication code	Antipsychotics: 1141202024, 1141153490, 1141195974, 1140867078, 1140867494, 1141171566, 2038459704, 1140872064, 1140879658, 1140867342, 1140867420, 1140882320, 1140872216, 1140910358, 1141200458, 1141172838, 1140867306, 1140867180, 1140872200, 1140867210, 1140867398, 1140882098, 1140867184, 1140867168, 1140863416, 1140909802, 1140867498, 1140867490, 1140910976, 1140867118, 1140867456, 1140928916, 1140872268, 1140867134, 1140867208, 1140867218, 1140867572, 1140879674, 1140909804, 1140867504, 1140868170, 1140879746, 1141152848, 1141177762, 1140867444, 1140867092, 1141152860, 1140872198, 1140867244, 1140868172, 1140867304, 1140872072, 1140879750, 1140868120, 1140872214, 1141201792, 1140882100, 1141167976
No diagnosis /medication	Exclusion from full cohort	BD cases		
		MD cases		
		SCZ cases		
		41270	ICD-10	F21, F29, F419, F410, F411, F418, F449, F444, F446, F440, F441, F448, F681, F688, F409, F400, F401, F402, F408, F341, F488, F489, F99, F601, F604, F607, F609
		41271	ICD-9	3000, 3001, 3002, 3004, 3005, 3009, 3012, 3015, 3016, 3019
		20002	Non-cancer illness code, self-reported	1286, 1287, 1288, 1289, 1290, 1291, 1531
		20003	Treatment/medication code	Antipsychotics (see above)<br>Antidepressants: 1140867820, 1140867948, 1140879616, 1140867938, 1140867690, 1141190158, 1141151946, 1140921600, 1140879620, 1141201834, 1140867152, 1140909806, 1140879628, 1140867640, 1141200564, 1141151982, 1140916288, 1141180212, 1140867860, 1140867952, 1140879540, 1140867150, 1140909800, 1140867940, 1140879544, 1140879630, 1140867856, 1140867726, 1140867884, 1140867922, 1140910820, 1140879556, 1141152732, 1140867920, 1140882244, 1140867852, 1140867818, 1141174756, 1140867916, 1140867888, 1140867850, 1140867624, 1140867876, 1141151978, 1140882236, 1140867878, 1201, 1140882312, 1140867758, 1140867712, 1140867914, 1140867944, 1140879634, 1140867756, 1140867934, 1140867960, 1140916282, 1141200570, 1141152736

Supplemental Table 6. Summary of characteristics of participants in the UK Biobank cohort who had no diagnosis of psychiatric disorders and were not on any antidepressants or antipsychotics

		Female		Male	
Total Number		148856		137306	
Average age at baseline in years (SD)		57 (7.9)		57.4 (8.1)	
Average BMI at baseline (SD)		26.7 (4.9)		27.7 (4.1)	
Current or previous smokers at baseline (%)		59328 (39.9)		68777 (50.1)	
		Case	Control	Case	Control
Incident AF	N (%)	6375 (4.3)	141290 (94.9)	11643 (8.5)	122363 (89.1)
	Age at baseline (SD)	62.7 (5.8)	56.7 (7.9)	62.3 (6.1)	56.8 (8.1)
Incident CAD	N (%)	3039 (2)	144506 (97.1)	8365 (6.1)	122328 (89.1)
	Age at baseline (SD)	61.9 (6.2)	56.9 (7.9)	60.6 (6.9)	56.9 (8.1)
Incident HF	N (%)	2958 (2)	145465 (97.7)	5938 (4.3)	130027 (94.7)
	Age at baseline (SD)	63.2 (5.7)	56.9 (7.9)	62.7 (6.1)	57.1 (8.1)
Hypercholesterolaemia status at baseline	N (%)	14309 (9.6)	121454 (81.6)	22797 (16.6)	93255 (67.9)
	Age at baseline (SD)	62.1 (5.7)	56 (7.9)	61.6 (6.3)	55.7 (8.2)
Hypertension status at baseline	N (%)	32739 (22.0)	101591 (68.2)	41542 (30.3)	76756 (55.9)
	Age at baseline (SD)	60.6 (6.7)	55.4 (7.9)	60.5 (6.9)	55.2 (8.2)
T2D status at baseline	N (%)	1694 (1.1)	141370 (95.0)	3556 (2.6)	123883 (90.2)
	Age at baseline (SD)	61.3 (6.6)	56.8 (7.9)	61.9 (6.1)	57.1 (8.2)

Supplemental Table 7. BioVU phenotype ascertainment criteria

Disease	Phecodes
AF	427.2
CAD	411.4
HF	428, 428.1, 428.2, 428.3, 428.4
BD	296.1
MD	296.2, 296.22
SCZ	295.1
Anxiety, dissociative and somatoform disorders	300
Personality disorders	301
Mood disorders	296

Supplemental Table 8. Sources of GWAS summary statistics used in PGS SNP weight estimation for UK Biobank and BioVU cohorts

Prediction cohort	Trait	Sample size	Ancestry	Reference
UK Biobank	AF	17931 cases and 115142 controls	Predominantly European	Christophersen et al. [55]
	BD	20352 cases and 31358 controls	European	Stahl et al. [56]
	CAD	60801 cases and 123504 controls	Predominantly European	Nikpay et al. [57]
	HF	40805 cases and 542362 controls	European	The full dataset (including UK Biobank individuals) is available in Shah et al. [58]). The dataset used in this study (with no UK Biobank individuals) is unpublished.
	MD	121198 cases and 329421 controls	European	The full dataset (including UK Biobank individuals) is available in Wray et al. [21]). The dataset used in this study (with no UK Biobank individuals) is unpublished.
	SCZ	40675 cases and 64643 controls	Predominantly European	Pardiñas et al. [59]
BioVU	BD	41917 cases and 371549 controls	European	Mullins et al. [60]
	MD	170756 cases and 329443 controls (excluding the 23andMe cohort)	European	Howard et al. [61]
	SCZ	53386 cases and 77258 controls	European	Trubetskoy et al. [62]

Supplemental Table 9. Prediction accuracy of PGSs

Prediction cohort	PGS	Number of SNPs	N_{cases}	$N_{controls}$	OR per SD increase of PGS (95% CI)	p-value	AUC
UK Biobank	AF	1002671	28634	316535	1.52 (1.50 - 1.54)	$< 2\times10^{-16}$	0.75
	BD	1008170	1402	286162	2.01 (1.90 - 2.12)	$< 2\times10^{-16}$	0.68
	CAD	1007431	24708	320461	1.59 (1.57 - 1.62)	$< 2\times10^{-16}$	0.77
	HF	1006789	14142	330528	1.25 (1.23 - 1.27)	$< 2\times10^{-16}$	0.74
	MD	1007847	20090	286162	1.33 (1.31 - 1.35)	$< 2\times10^{-16}$	0.61
	SCZ	987863	1387	286162	1.83 (1.74 - 1.94)	$< 2\times10^{-16}$	0.67
BioVU	BD	778307	1694	47363	1.31 (1.24 - 1.37)	$< 2\times10^{-16}$	0.61
	MD	770389	8942	40115	1.19 (1.16 - 1.21)	$< 2\times10^{-16}$	0.60
	SCZ	779202	230	48827	1.72 (1.49 - 1.99)	3.0×10^{-13}	0.67

Supplemental Table 10. Prediction accuracy of PGSs generated by SBayesR in the BioVU cohort

Prediction cohort	PGS	Number of SNPs	OR per SD increase of PGS (95% CI)	p-value	AUC
BioVU	BD	781964	1.32 (1.25 - 1.39)	$< 2\times10^{-16}$	0.61
	MD	800305	1.19 (1.16 - 1.22)	$< 2\times10^{-16}$	0.60
	SCZ	795253	1.81 (1.58 - 2.07)	$< 2\times10^{-16}$	0.69

Supplemental Figures

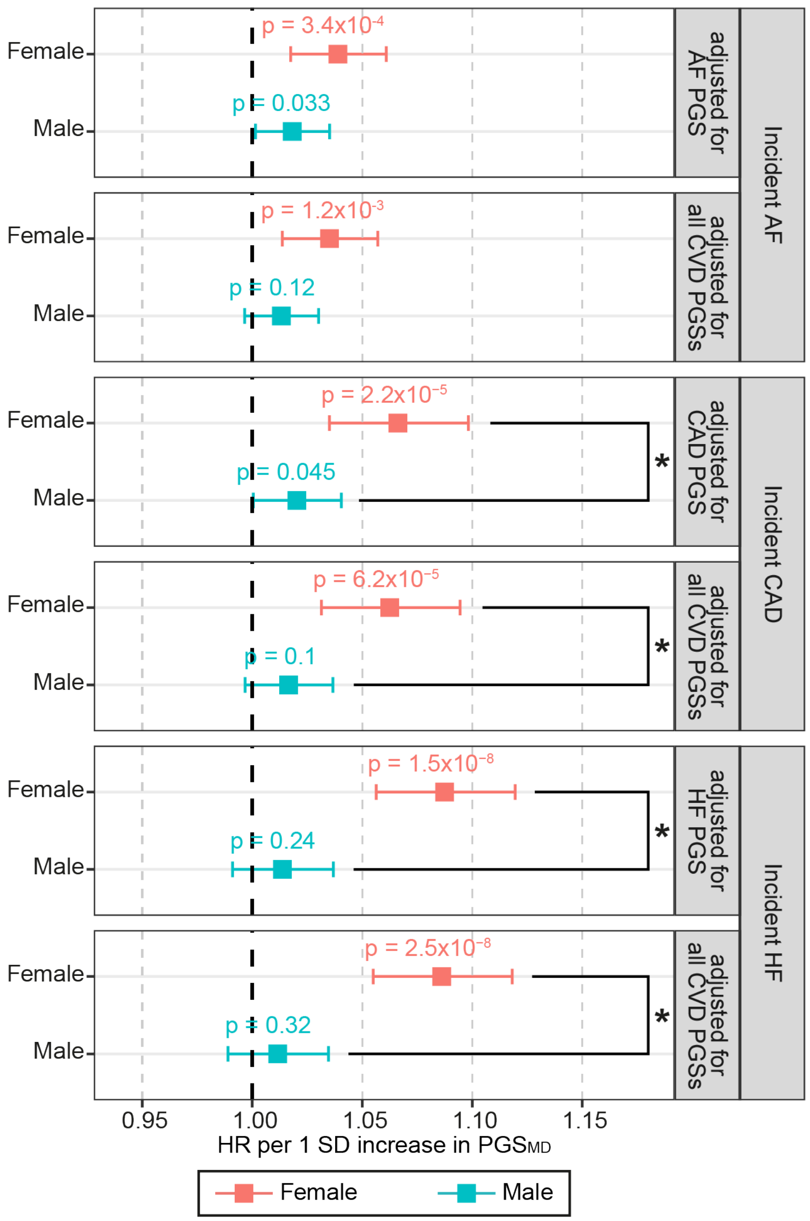

Supplemental Figure 1. Sensitivity analyses on the association between PGS_{MD} and incident CVDs, performed on the UK Biobank cohort, where the corresponding CVD PGS or all three CVD PGSs (AF, CAD and HF) were added as covariates to the model. Associations for the sex-stratified cohorts (red - female; blue - male) were estimated with Cox proportional hazards regression models, including CVD PGS(s), BMI, smoking status, genotyping array and 20 genetic PCs as covariates. X-axis shows the HR per SD increase in PGS, with p-values labelled, and error bars indicate 95% confidence intervals. Asterisk (*) indicates a statistically significant difference in the log(HR) values between females and males (two-sided Wald test $p<0.05$). Dark grey line indicates HR of 1. AF, atrial fibrillation; CAD, coronary artery disease; CVD, cardiovascular disease; HF, heart failure; HR, hazard ratio; MD, major depression; PGS, polygenic score; SD, standard deviation.

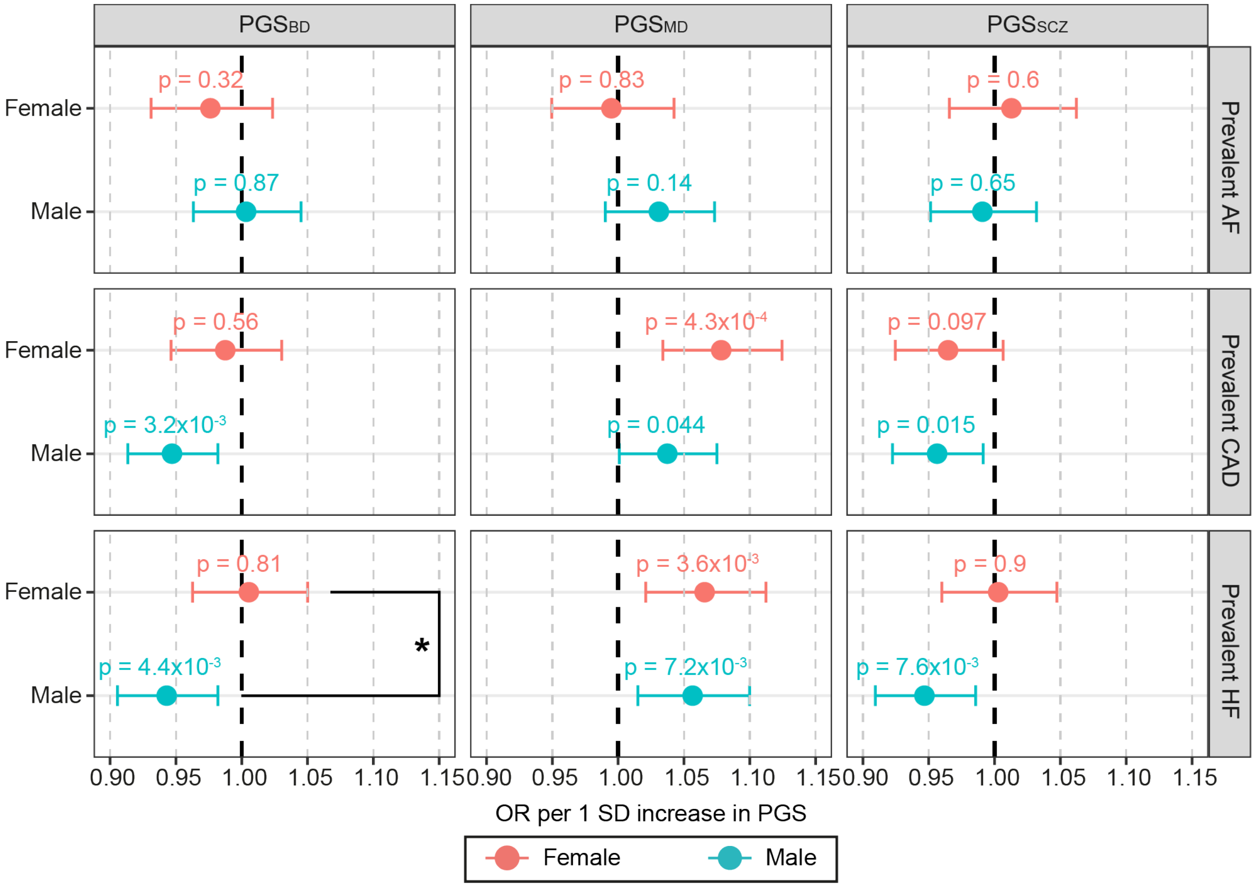

Supplemental Figure 2. Association between psychiatric disorder PGSs and prevalent CVDs in the BioVU cohort. Associations for the sex-stratified cohorts (red - female; blue - male) were estimated with logistic regression, including median age, median BMI, smoking status and 10 genetic PCs as covariates. X-axis shows the OR per SD increase in PGS, with p-values labelled, and error bars indicate 95% confidence intervals. Asterisk (*) indicates a statistically significant difference in the log(OR) values between females and males (two-sided Wald test $p<0.05$). Dark grey line indicates OR of 1. AF, atrial fibrillation; BD, bipolar disorder; CAD, coronary artery disease; HF, heart failure; MD, major depression; OR, odds ratio; PGS, polygenic score; SCZ, schizophrenia; SD, standard deviation.

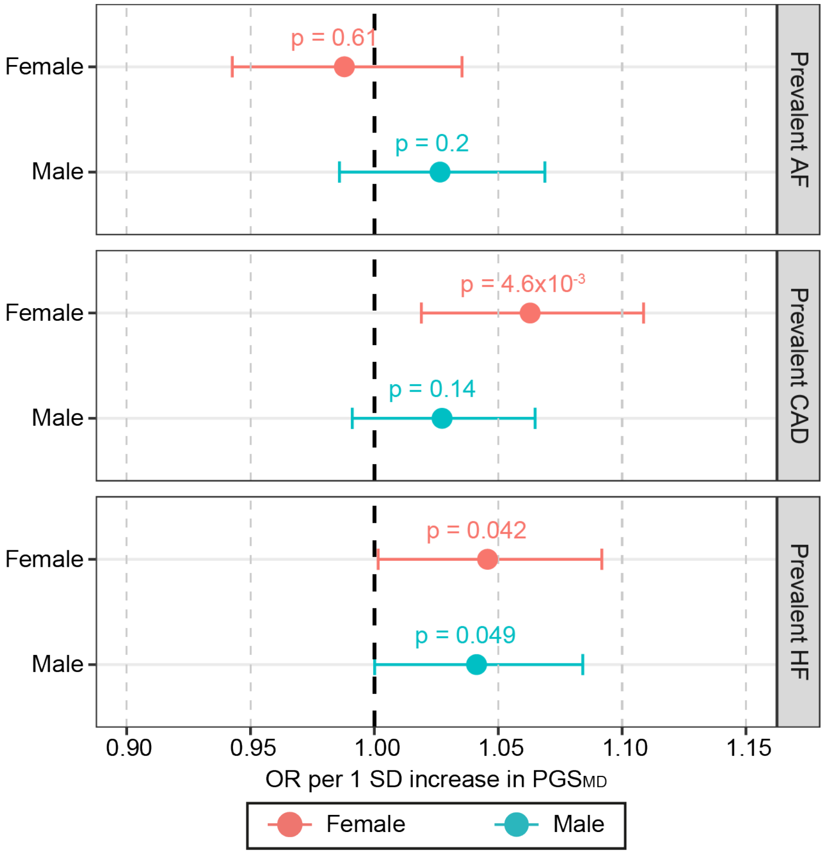

Supplemental Figure 3. Association between PGS_{MD} and prevalent CVDs amongst individuals of the BioVU cohort, where the status of psychiatric disorders and antidepressant were added as additional covariates to the model. OR was estimated with logistic regression, including the status of psychiatric disorders, antidepressant use, median age, median BMI, smoking status and 10 genetic PCs as covariates. X-axis shows the OR per SD increase in PGS, with p-values labelled, and error bars indicate 95% confidence intervals. Dark grey line indicates OR of 1. AF, atrial fibrillation; CAD, coronary artery disease; HF, heart failure; MD, major depression; OR, odds ratio; PGS, polygenic score; SD, standard deviation.

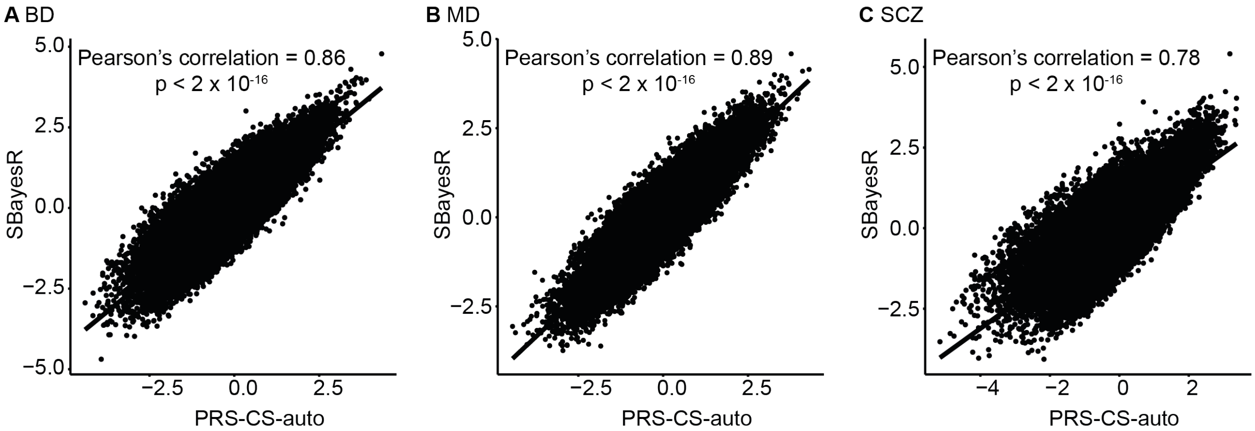

Supplemental Figure 4. Pearson's correlation between PGSs generated using PRS-CS-auto and SBayesR for (A) BD, (B) MD and (C) SCZ. BD, bipolar disorder; MD, major depression; SCZ, schizophrenia.
